# Evaluation of deep learning-based quantitative computed tomography for opportunistic osteoporosis screening

**DOI:** 10.1038/s41598-023-45824-7

**Published:** 2024-01-05

**Authors:** Sangseok Oh, Woo Young Kang, Heejun Park, Zepa Yang, Jemyoung Lee, Changwon Kim, Ok Hee Woo, Suk-Joo Hong

**Affiliations:** 1 Department of Radiology, Guro Hospital, Korea University Medical Center, Korea University College of Medicine, 148, Gurodong-ro, Guro-gu, Seoul, 08308 Republic of Korea; 2 ClariPi Inc., Seoul, Republic of Korea; 3 Department of Applied Bioengineering, Seoul National University, Seoul, Republic of Korea

**Keywords:** Diseases, Medical research

## Abstract

To evaluate diagnostic efficacy of deep learning (DL)-based automated bone mineral density (BMD) measurement for opportunistic screening of osteoporosis with routine computed tomography (CT) scans. A DL-based automated quantitative computed tomography (DL-QCT) solution was evaluated with 112 routine clinical CT scans from 84 patients who underwent either chest (N:39), lumbar spine (N:34), or abdominal CT (N:39) scan. The automated BMD measurements (DL-BMD) on L1 and L2 vertebral bodies from DL-QCT were validated with manual BMD (m-BMD) measurement from conventional asynchronous QCT using Pearson’s correlation and intraclass correlation. Receiver operating characteristic curve (ROC) analysis identified the diagnostic ability of DL-BMD for low BMD and osteoporosis, determined by dual-energy X-ray absorptiometry (DXA) and m-BMD. Excellent concordance were seen between m-BMD and DL-BMD in total CT scans (r = 0.961/0.979). The ROC-derived AUC of DL-BMD compared to that of central DXA for the low-BMD and osteoporosis patients was 0.847 and 0.770 respectively. The sensitivity, specificity, and accuracy of DL-BMD compared to central DXA for low BMD were 75.0%, 75.0%, and 75.0%, respectively, and those for osteoporosis were 68.0%, 80.5%, and 77.7%. The AUC of DL-BMD compared to the m-BMD for low BMD and osteoporosis diagnosis were 0.990 and 0.943, respectively. The sensitivity, specificity, and accuracy of DL-BMD compared to m-BMD for low BMD were 95.5%, 93.5%, and 94.6%, and those for osteoporosis were 88.2%, 94.5%, and 92.9%, respectively. DL-BMD exhibited excellent agreement with m-BMD on L1 and L2 vertebrae in the various routine clinical CT scans and had comparable diagnostic performance for detecting the low-BMD and osteoporosis on conventional QCT.

## Introduction

The world’s aged population is increasing widely. According to the World Health Organization, the world’s population of people aged 60 years and older will double to 2.1 billion by 2050^1^. Increased demand for medical imaging services with the aging population and other factors have increased the number of imaging examinations, including computed tomography (CT), over the past several decades^2,3^. Due to the desire to inhibit wasteful diagnostic imaging^4^ and demands for new approaches to add value to imaging, opportunistic CT has emerged. This is an approach to derive new biomarkers from routine CT examinations, such as scanning for osteoporosis and sarcopenia^5^.

Osteoporosis, which involves reduced bone mass and bone strength, leads to increased susceptibility to fragility fractures^6^. Although early detection of asymptomatic osteoporosis or osteopenia for prevention of osteoporotic fracture is considered a major health concern, osteoporosis is both an underdiagnosed and undertreated disease. Several reasons for this have been proposed in the United States^7^, including the declination of central dual-energy X-ray absorptiometry (DXA) usage, which is the gold standard for diagnosing osteoporosis^8–10^. However, DXA limitations include overestimation of bone mineral density (BMD) in patients with vertebral osteophytes or end plate sclerosis and abdominal aortic calcification, while quantitative computed tomography (QCT) is less affected^11,12^. Therefore, to increase osteoporosis detection without using DXA, the development of a precise and simple tool that can calculate BMD using opportunistic CT scans is required.

In previous studies, many attempts have focused on adopting artificial intelligence solutions to automatically and precisely evaluate QCT, increasing efficiency in image departments and resolving underdiagnosed rates of osteoporosis in the clinical field. Yasaka et al.^13^ developed a convolutional neural network (CNN) model predicting BMD of the lumbar vertebrae using axial CT images that showed a strong correlation with BMD derived from DXA.

The purpose of this study was to describe a new deep learning (DL) algorithm for vertebral segmentation and localizing of L1 and L2 vertebrae in routine clinical chest, abdomen, and lumbar spine CT scans and automatically draw a region of interest (ROI) from each vertebra to calculate volumetric QCT BMD. Also we evaluated diagnostic efficacy of the DL-based automated QCT BMD measurement for opportunistic screening of osteoporosis with routine clinical chest, lumbar spine, and abdominal CT scans.

## Results

### Patient characteristics

According to WHO guidelines, patients were divided into three groups based on central DXA value: osteoporosis (18 patients, 21.4%), osteopenia (38 patients, 45.2%), and normal (28 patients, 33.3%) (Table 1). The low-BMD group was defined as those with either osteoporosis or osteopenia and included 56 patients (66.7%). In terms of the 112 CT scans and based on central DXA values, 25 CTs (22.3%) were classified as osteoporosis, 51 (45.5%) as osteopenia, 76 (25 + 51) (67.9%) as low BMD, and 36 (32.1%) as normal.

**Table 1:** Patient characteristics.

	Chest CT set	Spine CT set	Abdomen CT set	Total set
*N*	39	34	39	112
Age (year)*	54 ± 9	72 ± 7	57 ± 11	61 ± 12
Population**	39	34	39	84
Male	4	11	2	17
Female	35	23	37	68
BMD(DXA)				
Osteoporosis	8	6	11	25
Osteopenia	17	20	14	51
Low BMD***	25	26	25	76
Normal	14	8	14	36

*Represents mean ± standard deviation.

**28 patients that underwent more than 2 different CT scans were accounted individually.

***Low BMD defined as osteoporosis or osteopenia.

Table 2 shows the overall and group-specific data classified as normal, osteopenia, or osteoporosis based on both DL-BMD and central DXA. Among the total 112 CT scans, there were 46 CTs classified as normal, 32 as osteopenia, 34 as osteoporosis, and 66 (32 + 34) as low BMD based on DL-BMD.

**Table 2:** Overall bone density distribution of total CT Scans based on DXA and DL-BMD.

	DL-BMD				
	Normal	Osteopenia	Osteoporosis	Low BMD*	Total
BMD(DXA)					
Normal	27	9	0	9	36
Osteopenia	15	19	17	36	51
Osteoporosis	4	4	17	21	25
Low BMD*	19	23	34	57	76
Total	46	32	34	66	112

*Low BMD defined as osteoporosis or osteopenia.

### Performance of DL-QCT

**Figure 1:**
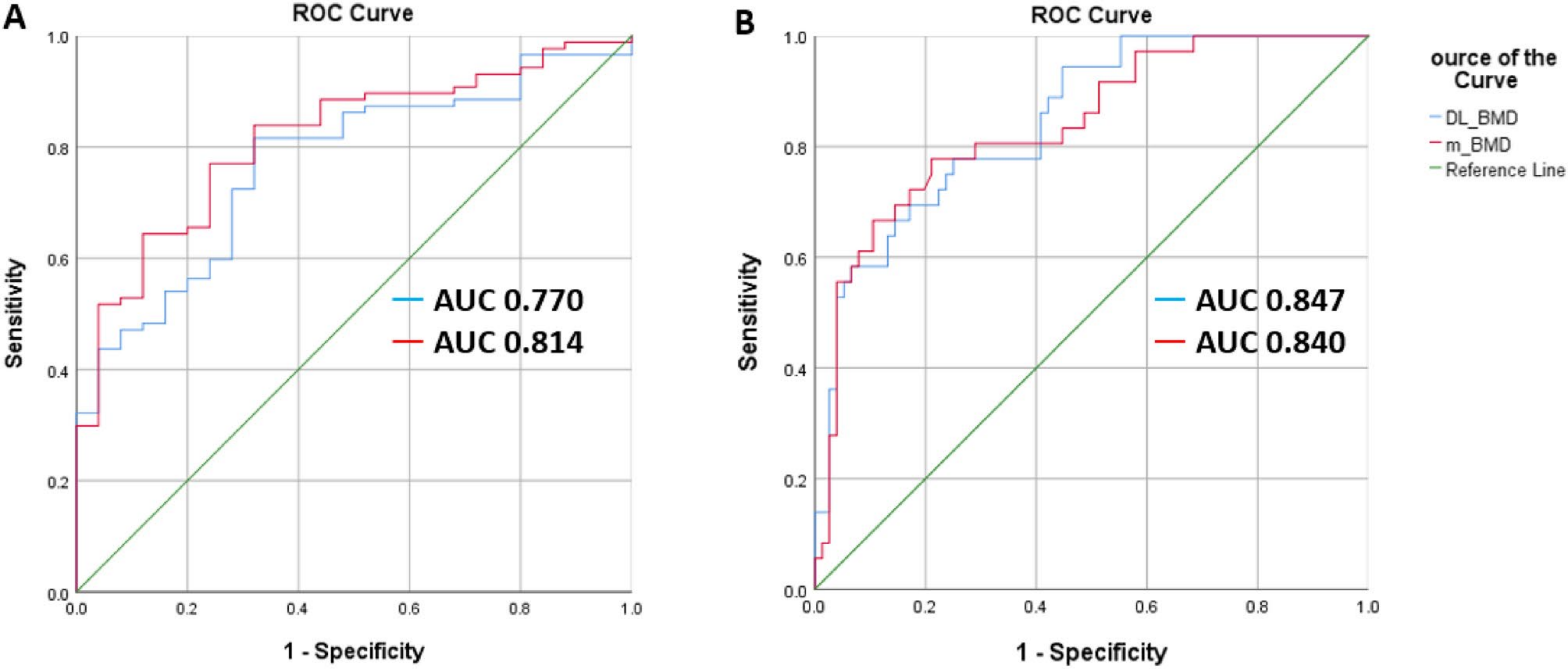
ROC curves of m-BMD and DL-BMD for diagnosis of osteoporosis and low BMD. (**A**, **B**). The AUC values were calculated for diagnosing osteoporosis (**A**) and low BMD (**B**) based on the central DXA. m-BMD = manual BMD, DL-BMD = deep learning-based automated bone mineral density, AUC = area under the receiver operating characteristic curve, Low BMD = osteoporosis or osteopenia, DXA = dual-energy X-ray.

**Figure 2:**
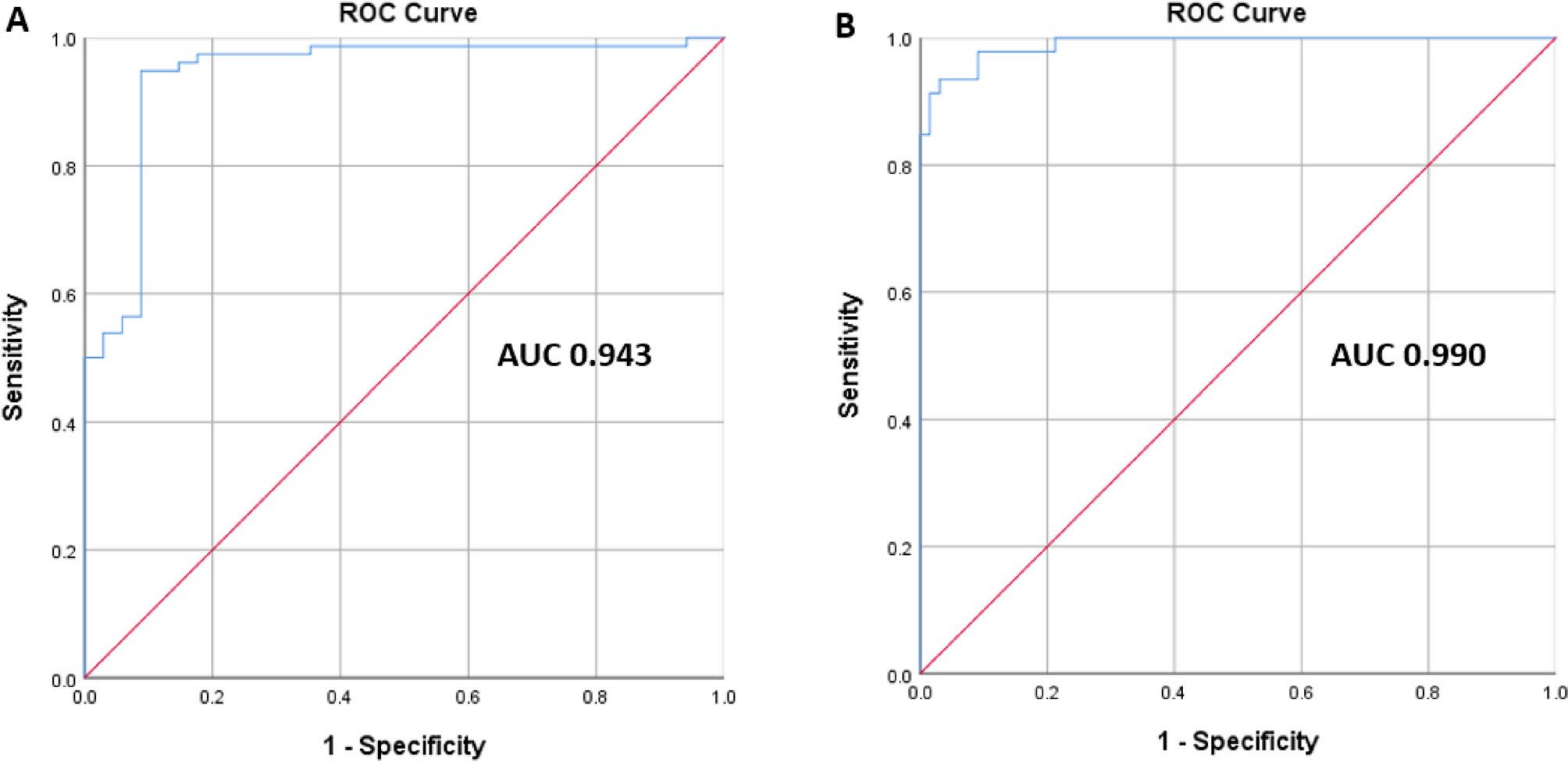
ROC curves of DL-BMD for diagnosing osteoporosis and low BMD. (**A**–**C**) The AUC values were calculated for diagnosing osteoporosis (**A**) and low BMD (**B**) based on m-BMD values. DL-BMD = deep learning-based automated bone mineral density, m-BMD = manual BMD, AUC = area under the receiver operating characteristic curve (AUC), Low BMD = osteoporosis or osteopenia a.

When comparing the m-BMD and DL-BMD values (Table 3), the Pearson coefficient was 0.961 (*p* < 0.001) and ICC absolute value was 0.979 (*p* < 0.001). The sensitivity, specificity, and accuracy for osteoporosis based on central DXA of m-BMD were 68.0%, 80.5%, and 77.7% respectively, and those for low BMD were 76.3%, 77.8%, and 76.8% (Table 4). The sensitivity, specificity, and accuracy for osteoporosis based on central DXA of DL-BMD were 68.0%, 80.5%, and 77.7%, respectively, and those for low BMD were 75.0%, 75.0%, and 75.0%, similar to m-BMD (Table 4). In ROC analysis, the AUC values of m-BMD for osteoporosis and low BMD were 81.4% (95% CI 73.0–88.1%) and 84.0% (95% CI 75.8–90.2%) respectively. The respective AUC values of DL-BMD for osteoporosis and low BMD were 77.0% (95% CI 68.1–84.4%) and 84.7% (95% CI 76.7–90.8%) and were slightly higher than those of m-BMD for low BMD, although the difference was not statistically significant (Fig. 1). Diagnostic performance of DL-BMD considering the m-BMD values instead of central DXA values also was evaluated. The sensitivity, specificity, and accuracy for osteoporosis based on m-BMD of DL-BMD were 88.2%, 94.5%, and 92.9%, respectively and those for low BMD based on m-BMD were 95.5%, 93.5%, and 94.6% (Table 4). In ROC analysis, the AUC values of DL-BMD for osteoporosis and low BMD based on m-BMD were 94.3% (95% CI 88.2–97.8%) and 99.0% (95% CI 94.9–100.0%), respectively (Fig. 2).

**Table 3:** Correlation of DL-BMD and m-BMD in total CT scans.

	Testing Cohorts			
	Chest CT	Spine CT	Abdomen CT	Total
Pearson coefficient	0.929	0.904	0.989	0.961
ICC-absolute	0.964	0.939	0.995	0.979

**Table 4:** Diagnostic performances of m-BMD and DL-BMD.

	m-BMD versus DXA	DL-BMD versus DXA	DL-BMD versus m-BMD
Sensitivity			
Osteoporosis	0.680	0.680	0.882
Low BMD	0.763	0.750	0.955
Specificity			
Osteoporosis	0.805	0.805	0.945
Low BMD	0.778	0.750	0.935
Accuracy			
Osteoporosis	0.777	0.777	0.929
Low BMD	0.768	0.750	0.946

## Discussion

The DL-BMD showed excellent agreement with m-BMD in mean measurements and strong positive correlation and excellent concordance was seen between m-BMD and DL-BMD based on Pearson correlation and absolute ICC. These results correlate well with a previous study^17^ that compared BMD values derived from DL models using axial CT scans to manually derive QCT values.

The m-BMD showed relatively low diagnostic performance by setting the central DXA value as the baseline value. However, even DXA has limitations such as overestimation in spondylosis since QCT is less affected. In certain aspects, QCT might be a more accurate method than BMD, so the diagnostic performance of m-BMD based on central DXA BMD does not mean that QCT is an inappropriate method to measure bone density.

**Figure 3:**
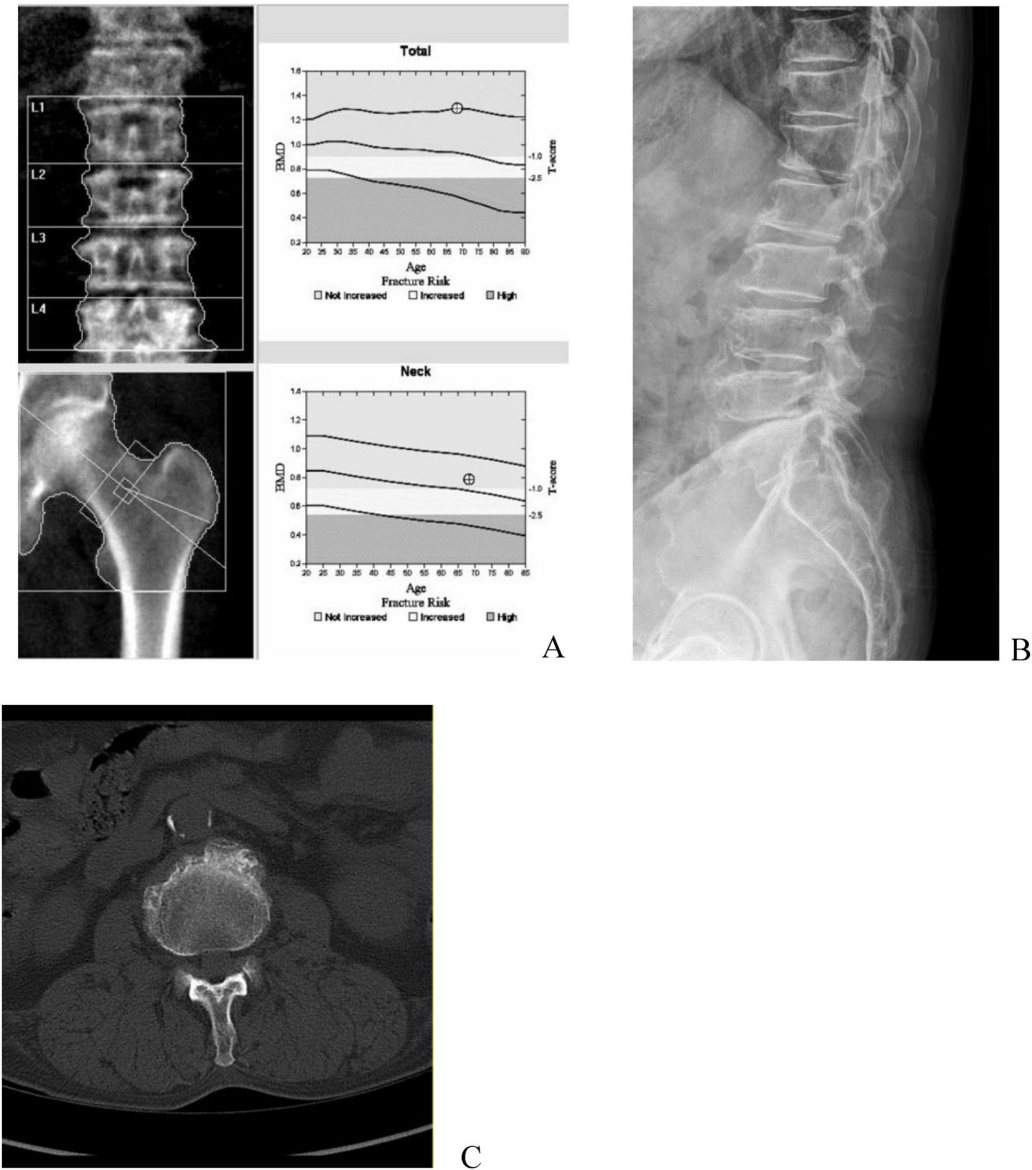
Images obtained from a 68-year-old man who was diagnosed via DXA to have normal CT scans (**A**–**C**). (**A**) T-scores for lumbar DXA, femoral neck, and total hip were 1.4, − 0.5, and − 0.5, respectively. The trabecular BMD of L1-L2 was 77.125 mg/cm^3^, showing a diagnosis of osteoporosis according to the American College of Radiology (ACR) guidelines. A lateral lumbar spine radiograph (**B**) and an axial CT image (**C**) depicted severe osteophytes and end plate sclerosis of the lumbar vertebrae. Abdominal aortic calcifications also were noted.

The diagnostic performance of DL-BMD when setting the m-BMD value as the standard value showed a higher tendency than when setting the central DXA value as the standard value. From a total of 112 CT scans, 19 (17.0%) showed osteoporosis by m-BMD but not by central DXA. Of these, 14 scans were classified the same using the DL-BMD and the m-BMD. In addition, 16 scans were spinal CT scans and all 19 (100%) scans had vertebral osteophytes or end plate sclerosis (Fig. 3), 18 (94.7%) had facet arthrosis, two (10.5%) had a bony island or focal sclerosis, and five (26.3%) had abdominal aortic calcification (AAC). It is well known from many studies^18–21^ that patients with bone degenerative changes such as facet joint degeneration, vertebral body osteophytes, and AAC may lead to overestimating BMD values using DXA. Li et al.^12^ reported that 41/140 patients (29.3%) were diagnosed with osteoporosis only by QCT and not by DXA. Yoon et al.^22^ reported that QCT identified osteoporosis in 30/59 patients (50.8%) that were not diagnosed with osteoporosis in DXA, which aligns with the results in this study. Thus, the results in this study show higher detection ability of QCT in osteoporosis patients having factors of BMD overestimation based on DXA results.

However, eight (7.1%) scans from five patients were classified as osteoporosis on central DXA but not on m-BMD. All eight scans had central DXA values classified as osteoporosis only at the femur neck, while the central DXA values of L-spines were classified as osteopenia or normal. In addition, classification of central DXA values on L-spines at each scan coincided with the classification based on m-BMD values. Additionally, when producing the ROI in the selected slice of the localized L1 or L2 vertebra, there could have been areas containing degenerative changes of the spine and showing abnormal HU values compared to the remainder of the spine. If algorithms are developed to identify abnormal areas representing degenerative changes based on HU values, it will increase the accuracy of the model. To make this feasible, the normal HU value should be definable, as related to various factors. In reference to a prior study^23^ showing the mean L1 attenuation of patients in abdominal and chest CTs with the effects of intravenous contrast agent and age, the normal value or ‘baseline’ HU value would be definable in CT scans used in this study. Studies for additional scanning parameters affecting the HU values of the bony trabeculae are needed in the future.

Even though we considered the effect of contrast agent using the HU-BMD conversion model, several recent studies have discussed the effects of the amount of contrast agent and the time interval between contrast agent injection and scanned time on volumetric BMD^24,25^. Therefore, factors that represent contrast should be added to the linear regression model in future study.

There are several strengths in this study. While previous studies determined the category of BMD based on HU values in opportunistic CT scans^26^, this study developed a model converting HU values into BMD values after localization and segmentation using DL techniques. Consistent with a previous study^17^, this study developed a HU-BMD conversion model with parameters including kVp.

Several limitations are noted. First, the DL model was evaluated using data from a single CT scanner in a small population size. Thus, our next study will include large datasets from various vendors and multi-centers. Second, this model has limited performance on patients having normal range BMD at the spine but abnormal at the pelvis. In our study, four CT scans were diagnosed as osteoporosis based on central DXA value of the femur neck and not on L-spines.

In conclusion, the DL-BMD exhibited excellent agreement with m-BMD on L1 and L2 vertebrae in the various routine clinical CT scans, with good diagnostic performance for detecting low BMD and osteoporosis determined with central DXA and m-BMD. Therefore, our study demonstrates that DL-based automated BMD measurement is feasible for opportunistic screening of low BMD and osteoporosis in various routine clinical CT scans.

## Methods

### Study population

**Figure 4:**
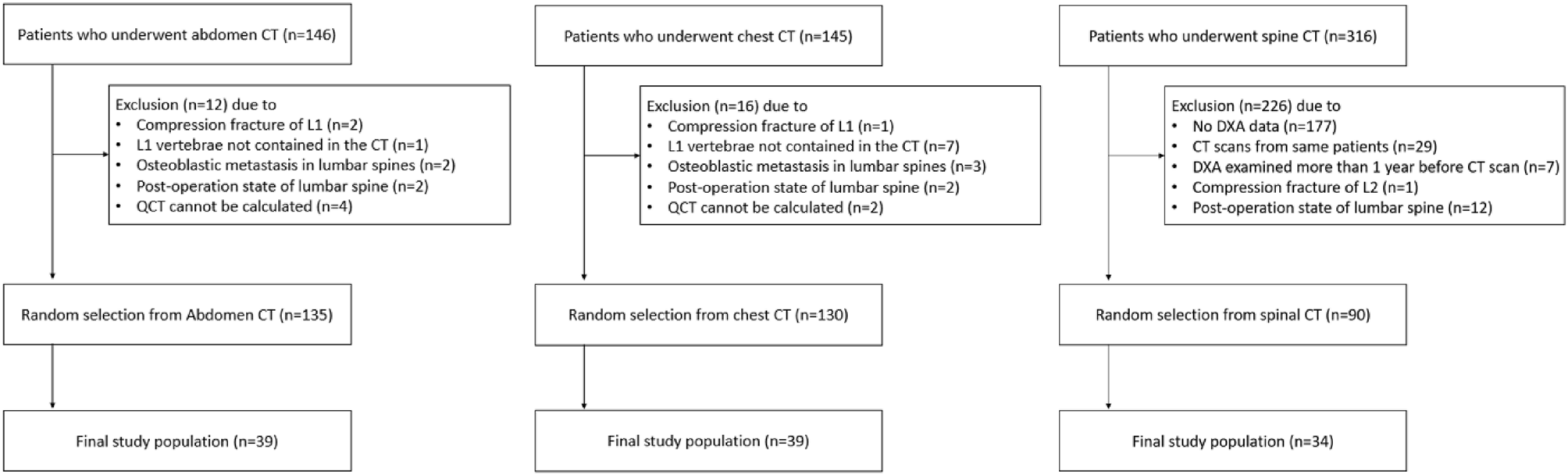
Flow diagram of the study patients. QCT = quantitative computed tomography, DXA = dual-energy X-ray absorptiometry.

The study was conducted in accordance with the Declaration of Helsinki (as revised in 2013). This retrospective study was approved by the medical ethics committee of Guro Hospital of Korea University (2021GR0370). Informed consent was not required by medical ethics committee of Guro Hospital of Korea University due to the retrospective nature of the study.and all clinical data was anonymized during analysis to maintain the patient privacy. And all methods were performed in accordance with the relevant guidelines and regulations of the committee of Guro Hospital of Korea University. Consecutive chest (146) and abdominal (145) CT images from February 2021 to March 2021 and spinal (316) CT images from October 2020 to July 2021 were collected at one tertiary hospital. All images were acquired with a single-vendor 192-channel dual-source CT scanner (SOMATOM Force, Siemens Healthcare, Erlangen, Germany). The scanning parameters in each CT protocol are provided in the S1 Table. Inclusion criteria were (1) routine clinical chest, abdominal, or spinal CT examination and (2) available DXA data within 1 year from the CT examination date. Exclusion criteria were (1) absence of L1 vertebrae in the CT scan, (2) operation at the L1 or L2 vertebrae, or (3) compression fracture or osteolytic/sclerotic lesion at the L1 or L2 vertebra that led to limited evaluation of volumetric bone mineral density (v-BMD). Finally, 84 patients (17 men and 68 women; mean age of 61 ± 12 years; age range, 33–82 years) and 112 CT scans satisfied all criteria and provided 39 CT scans each from the chest and abdomen and 34 spinal CT scans (Fig. 4; Table 1).

### Central DXA measurement

Central DXA values were measured using a Hologic Horizon W (S/N 301367 M) scanner (Hologic Inc., Bedford, MA, USA) with APEX 13.6.0.5 software (Hologic Inc.). L1 to L4 vertebrae and the left or right hip were scanned with the patient in the supine position. The T-score for L1–L4 and for the femoral neck plus the total hip measurement by DXA were used to diagnose osteoporosis. In concordance with the guidelines from the WHO^14^, osteoporosis, osteopenia, and normal were defined based on T score ≤ − 2.5 SD, between 2.5 SD and − 1.0 SD, and − 1.0 SD ≤ , respectively. Low BMD was defined as diagnosis of osteoporosis or osteopenia based on central DXA.

### Conventional QCT BMD measurement

**Figure 5:**
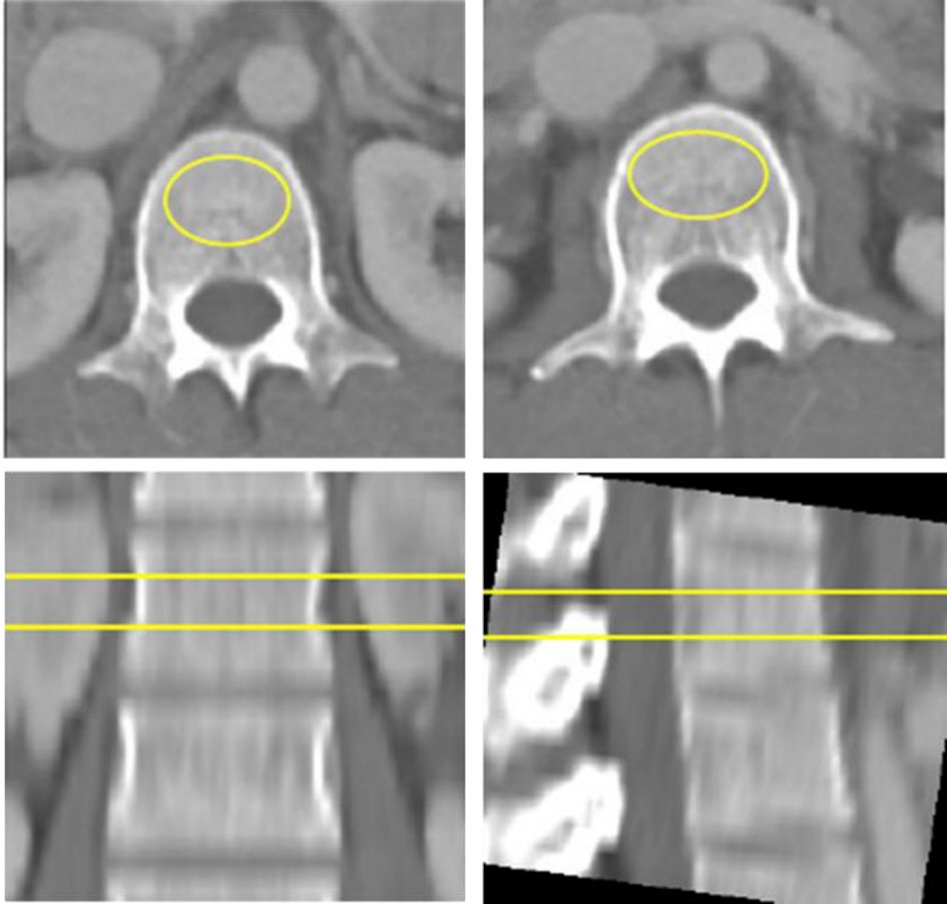
Manually measuring v-BMD on abdominal CT using asynchronous QCT. v-BMD = volumetric bone mineral density, QCT = quantitative computed tomography.

All routine clinical CT images were post-processed using QCT Pro Version 2 (Model 4 CT Calibration Phantom; MindWays Software Inc., Austin, TX, USA) for asynchronous measurement of QCT BMD. In concordance with the guidelines from the American College of Radiology (ACR)^15^, osteoporosis, osteopenia, and normal were defined as v-BMD < 80 mg/cm^3^, 80–120 mg/cm^3^, and > 120 mg/cm^3^, respectively. Figure 5 shows the process for measuring v-BMD manually on abdominal CT using asynchronous QCT. The ROI was drawn to include the trabecula as much as possible while avoiding the cortical bone and basivertebral vein.

### DL-BMD software development

The DL model was developed using the Python programming language (version 3.8.5; Python Software Foundation, Beaverton, OR, USA) and the DL framework of Google TensorFlow-GPU (version 2.4.0; Google, Mountain View, CA, USA) with Visual Studio Code (version 1.73.1, Microsoft Corporation, Redmond, WA, USA). The DL-based Automatic Bone Mineral Density (DL-BMD) measurement model consists of two stages, (1) lumbar spine segmentation and localization and (2) ROI and BMD calculation.

#### Lumbar spine segmentation and localization

##### Lumbar spine segmentation

We designed a U-Net-based segmentation network for spinal segmentation with additional field of view (FOV) augmentation and CT denoising for robustness in heterogeneous scan settings. Only the lumbar spine was segmented for L1 localization and BMD measurement.

##### Training and validation datasets

A total of 81 chest and abdominal CT scans from the VerSe2020 open data set and 34 spinal CT scans from the Philips Brilliance 64, iCT 256 and IQon, Philips Medical Care, Siemens SOMATOM Definition AS and AS + Siemens Healthineers were used. From the CTSpine1K open data set, 39 non-enhanced chest CT images with COVID-19 infections and 110 cases of contrast enhanced abdominal CT images from the 10th Medical Segmentation Decathlon (MSD T10) were used. A total of 264 cases (109,142 slices) of axial reconstruction CT images with paired binary spine mask from two major public datasets (VerSe2020 and CTSpine1K) was used for training (70%, 76,399 slices) and validation (30%, 32,743 slices) datasets for lumbar spine segmentation.

##### Model training

**Figure 6:**
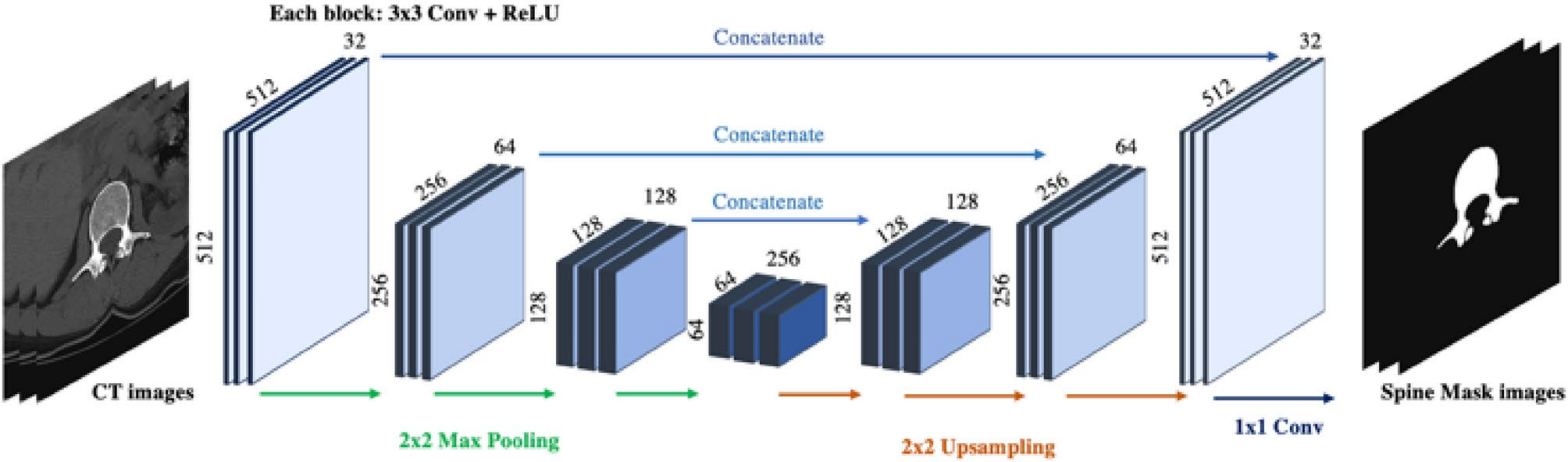
Overview of the 2D U-Net architecture with an input image matrix size of 512 × 512 and multiple layers. Arrows of different colors indicate different operations. Conv = Convolutional layer, ReLU = Rectified Linear Unit.

An encoder module and a decoder module with a concatenated skip-connection were utilized in the U-Net network, including a 3 × 3 kernel size convolution layer, rectified linear unit activation (ReLU), and a 2 × 2 max-pooling layer of the encoder module. With exception of the max-pooling layer being replaced by a 2 × 2 up-sampling layer with nearest-neighbor interpolation, the decoder module was nearly identical to that of the encoder. A 1 × 1 convolution layer was applied as the final layer to reduce feature dimensions (Fig. 6).

Pre-processing included several steps. First, all images were processed using window level (WL) and window width (WW) [300, 850]. Images were adjusted accordingly from -125 to + 725 HU and normalized from 0 to 1. With paired spinal mask data, only the lumbar spine mask was designated as 1; the other was 0. Several types of data augmentation methods, denoising techniques, and FOV adjustments were applied to acquire generalized learning and vendor-agnostic spine segmentation performance in heterogeneous scan settings. FOV augmentation was performed from the paired spinal mask data. Center of mass coordinates were calculated from the binary mask, and a boundary was drawn for the spine mask. We randomly scaled the width and height of the boundary box but always included the spinal area. The randomly scaled area was rescaled into a 512 × 512 size, and the same rescaling method was applied to paired CT images.

#### ROI production and BMD calculation

##### ROI production

**Figure 7:**
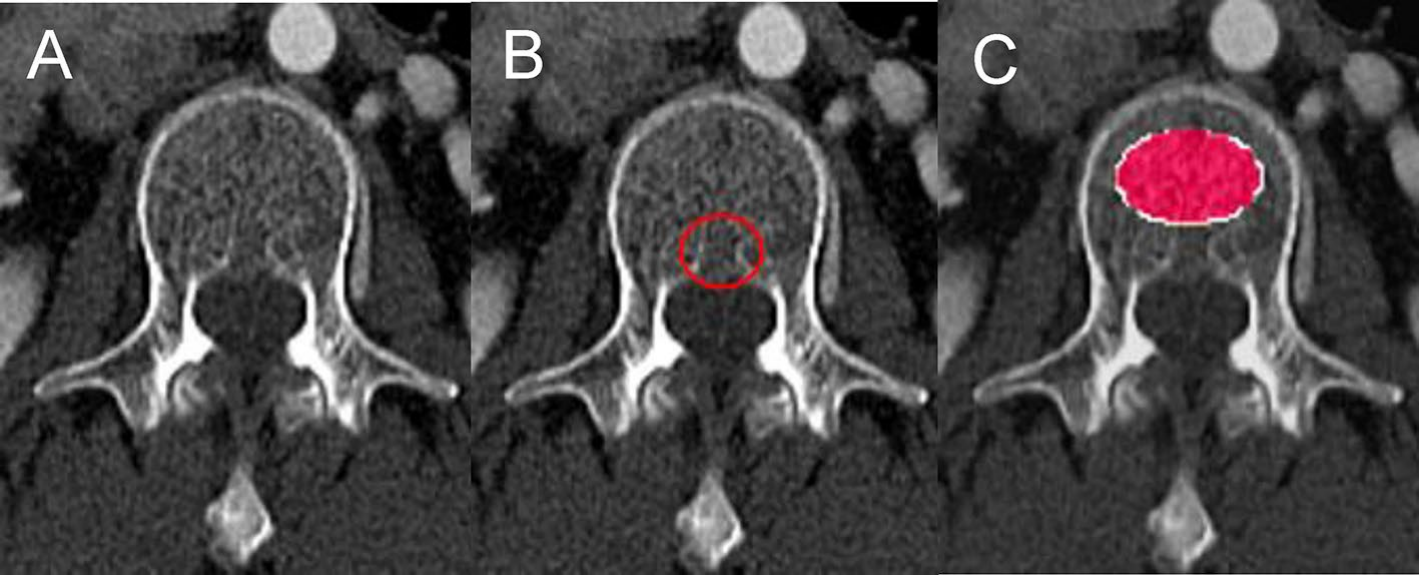
Process of DL-BMD measurement on chest CT. (**A**–**C**) The selected slice shows the raw data (**A**), area of basivertebral vein (**B**), and the ROI drawn (**C**) excluding the basivertebral vein (**B**) area. DL-BMD = deep learning-based automated bone mineral density, ROI = region of interest.

The ROI placement algorithm was performed using Python, OpenCV (version 4.5.3; Intel, Santa Clara, CA, USA) with the Visual Studio Code. The elliptical ROI was programmed to only encompass the inner trabecular bone region avoiding the cortical bone. To prevent inclusion of the basivertebral vein section, the ROI was programmed to produce 3–5 pixels anteriorly from the vein (Fig. 7). Three slices including L1 and L2 were selected individually for BMD measurement. The ROI was drawn on the slice closest to the center of the localized vertebrae, and the Hounsfield unit (HU) values were calculated.

##### HU-BMD conversion

Measurement of HU deviation and its impact on BMD was performed in advance following the BMD calibration in accordance with the kernel using the European Spine Phantom (QRM-ESP-04 model, hereinafter ESP). As a standard phantom, the L1, L2, and L3 areas in the ESP were filled with calcium hydroxyapatite to bone densities of 50, 100, and 200 (mg/cc), respectively. Regression analysis calibration was used to determine the HU value for each BMD value in the images acquired by ESP. The average HU values of the L1 and L2 positions were applied to the BMD calibration equation in accordance with the kVp and kernel to determine the final BMD value.

Based on a prior study by Perez et al.^16^, the L1 trabecular bone pre-contrast attenuation (${B\_HU}_{Pre}$) and post-contrast attenuation (${B\_HU}_{Post}$) were presented as a linear equation of ${B\_HU}_{Pre}$=0.87×${B\_HU}_{Post}$ (${r}^{2}$=0.72; *p* =  < 0.001). The HU-BMD conversion model used this equation, which reflects the effect of contrast agent.

### Performance evaluation of DL-BMD by automated QCT

The automated volumetric BMD measurements (DL-BMD) on L1 and L2 vertebral bodies from DL-QCT software were validated with manually measured volumetric BMD (m-BMD) from a conventional asynchronous QCT using Pearson’s correlation and absolute intraclass correlation coefficient (ICC). The diagnostic capability of DL-BMD for DXA-determined and m-BMD-determined low-BMD (including both osteoporosis and osteopenia) group and osteoporosis group was assessed using receiver operating characteristic (ROC) analysis. For comparison of the diagnostic performance between the two groups, the Delong test was used. Performance evaluation was conducted for the total dataset along with chest, spine, and abdominal CT sub-groups based upon 2 × 2 contingency tables obtained by one-sided comparison between the results of the DL-BMD test and central DXA and between those of DL-BMD and m-BMD. SPSS Statistics (version 25.0, IBM Corp., Armonk, NY, USA) and MedCalc (Version 20.217, MedCalc Software, Ostend, Belgium) were used for statistical analysis. A *p* value < 0.05 was considered significant.

### Supplementary Information

Supplementary Information.

## Data Availability

The datasets generated during and/or analysed during the current study are not publicly available due to privacy of patient information, but are definitely available from the corresponding author if requested. All requests are greeted.
