# Supplementary material for: Evaluation of deep learning-based quantitative computed tomography for opportunistic osteoporosis screening
Source: Sci Rep. 2024 Jan 5;14:363. doi: 10.1038/s41598-023-45824-7 (PMC10770031; doi:10.1038/s41598-023-45824-7)
Supplement: Supplementary file 1 — Supplementary Information. [file 41598_2023_45824_MOESM1_ESM.pdf]

Original Research

Evaluation of Deep Learning-Based Quantitative Computed Tomography for Opportunistic Osteoporosis Screening

Sangseok Oh, Woo Young Kang, Heejun Park, Zepa Yang, Jemyoung Lee, Changwon Kim, Ok Hee Woo[#], Suk-Joo Hong[#]

Department of Radiology, Guro Hospital, Korea University Medical Center, Seoul, Republic of Korea (S.O., W.Y.K., H.P., Z.Y., O.H.W., S.-J.H.)

ClariPi Inc., Seoul, Korea (J.L., C.K.)

Department of Applied Bioengineering, Seoul National University, Seoul, Republic of Korea (J.L.)

Corresponding Author:

Ok Hee Woo, MD, PhD

E-mail: wokhee@korea.ac.kr

Suk-Joo Hong, MD, PhD

Department of Radiology, Guro Hospital, Korea University College of Medicine,

148, Gurodong-ro, Guro-gu, 08308 Seoul, South Korea

E-mail: hongsj@korea.ac.kr

TEL: 82-02-2626-1341

FAX: 82-02-863-9282

# Ok Hee Woo and Suk-Joo Hong contributed equally to this work and should be considered co-first authors.

Supplementary Material

S1 Table. CT scanning parameters used in each protocol

CT protocol	Tube voltage (kVp)	Tube current (mA)	Collimation (mm)	Pitch	Kernel	Section thickness (mm)	Contrast (M, ml/sec)
Abdomen CT	100	automatic	0.6	0	Admire Br40d/2	5	300, 3
Chest CT	90	automatic	0.6	0.7	Admire Br54d/2	3	300, 4
Lumbar spine CT	100	automatic	0.6	0	Admire Br59d/2	3	None
